# Knowledge and views of physicians and nurses about physical activity advice in oncology care: a cross-sectional study

**DOI:** 10.11604/pamj.2022.41.57.30121

**Published:** 2022-01-20

**Authors:** Youness Azemmour, Saber Boutayeb, Said Nafai, Amine Souadka, Hind Mrabti, Ibrahim Elghissassi, Abdelghafour Marfak, Hassan Errihani

**Affiliations:** 1 Clinical Research Biostatistics and Epidemiology Laboratory, Faculty of Medicine and Pharmacy, Mohammed V University, Rabat, Morocco,; 2 Translational Oncology Research Team, National Institute of Oncology, Ibn Sina University Hospital Center, Mohammed V University, Rabat, Morocco; 3 Division of Occupational Therapy, School of Health Sciences, American International College, USA,; 4 National School of Public Health, Rabat, Morocco

**Keywords:** Physical activity, advice, healthcare professionals, oncology care

## Abstract

**Introduction:**

the benefits of physical activity have been approved in oncology care. This is why healthcare professionals must play a principle role in promoting physical activity during all cancer care pathway. The purpose of this study was to explore and compare physicians' and nurses' knowledge and views toward physical activity advice in oncology care.

**Methods:**

this cross-sectional study included Moroccan physicians and nurses specialized in oncology. Participants were asked to complete an anonymous questionnaire. The inferential statistics were performed to find a difference between physicians' and nurses' knowledge and views.

**Results:**

questionnaires were returned by 154 healthcare professionals (response rate 48. 6%). The majority was informed about the physical activity benefits in oncology. The physicians seem to be more informed than nurses about physical activity benefits in oncology (Chi-squared test, p=0.016). The majority thought that physical activity is beneficial in post-treatment (59.7%), while 24% only granted these benefits in the palliative care. The Participants expressed positive views about physical activity in oncology, especially nurses who seem to agree the most with implementation of a physical activity program in the hospital (Mann-Withney, p=0.04). The majority of participants stated that there are some clinical factors related to the patient that constitute a barrier of physical activity advising.

**Conclusion:**

the lack of knowledge, self-declared by the majority of participants, underlines the need to strengthen training actions about physical activity advice in health professionals, especially nurses for people with cancer.

## Introduction

In the world, cancer is considered as a public health problem. In 2017, there were 24.5 million incident cancer cases, with 9.6 million cancer deaths. Also, in 195 countries from 1990 to 2017, the cancer burden has been estimated at 233.5 million years adjusted for disability, of which 97% of years of life lost and 3% of years lived with a disability [1]. Despite the progress made in oncology in saving lives for patients with cancer (PC), the oncological approach alone remains insufficient to provide a response to the cancer functional impact for the cancer survivor (CS). Therefore, it is necessary to have a global approach to health care, in which the physical activity (PA) advice must be integrated. In fact, there is strong scientific evidence to support the PA benefits on physical functions and quality of life for patients living with cancer [2]. However, the majority of CS don´t maintain a sufficient level of PA to achieve these benefits [3].

Oncology healthcare professionals (HCPs) are in a good position to promote PA, given the trust they have from their patients and the multiple contacts they have with them during all cancer care pathway. As a result, they are able to give encouragement strategies for their patients [4]. Therefore, they can participate in changing patient attitudes and behaviors through brief but effective conversations [5]. HCPs seem to have favorable views about PA in oncology care. Also, they want more training opportunities in this field to feel able to advice PA for their patients [6-8]. The favorable HCPs´ views on PA in oncology care has a good influence on their attitude towards the PA advice for their patients. Indeed, the high PA advice rate has been associated with the favorable HCPs´ views on PA [9].

In Morocco, Cancer is a major concern. However, in the National Cancer Prevention and Control Plan 2020-2029, the promotion of PA is reduced to the primary prevention. It does not yet occupy the place it deserves in the all cancer care pathway. In reality, this observation could be generalized to several similar countries. Many studies around the world have explored the knowledge and views both of physicians and nurses, on the PA advice in oncology, but few of them have compared their knowledge and views. However, it has been shown that some HCPs´ socio-professional characteristics are associated with their knowledge about the PA benefits in cancer care pathway, such as experience [10] and specialty in oncology [11].

Being the first study - to the authors' knowledge - about this topic in Morocco, we aim to achieve a collective awareness of the PA advice in oncology care. This study aimed to explore and compare physicians´ and nurses´ knowledge and views toward PA advice in oncology care.

## Methods

**Study design:** this research is a cross-sectional study which focused on the HCPs´ knowledge and views about PA advice in oncology care.

**Setting:** the participants are made up of specialized physicians and nurses in oncology working in the National Institute of Oncology at the Ibn Sina University Hospital in Rabat. It is considered the main center of oncology care and the national reference center for the management and care of cancer in Morocco. The data collection was carried out between the months of February and November 2020. Given the restrictions linked to the COVID-19 pandemic, the questionnaire was administrated via paper and then by electronic version. A reminder was systematically sent if the questionnaire was not returned within four weeks. Completing the questionnaire takes an average of 7 to 10 minutes. Participants were asked to complete the questionnaire individually in order to avoid peer influence.

**Participants:** this study included HCPs clinicians (physicians and nurses) who care for PC and CS and have regular contact with them. A total of 317 participants were identified from institutional sources (61 for physicians and 256 for nurses). The exhaustive sampling was chosen as the sampling method, so all participants were asked to complete an anonymous questionnaire.

**Study instrument:** the questionnaire is organized in three sections: a) demographic and professional characteristics of the participants; (b) knowledge of the PA benefits in oncology; (c) views on PA advice in oncology. The last section is composed by ten items scored using Likert scale, which contain five degrees of agreement (strongly agree, agree, indifferent, disagree, strongly disagree). These items are interested in: a) the views of physicians and nurses about the PA benefits in oncology; (b) the patients´ perception on PA in oncology care; (c) the patients' adherence to the PA advice given by HCPs and the role played by family and friends; (d) the need for more information in order to advice PA and the clinical factors that prevent HCPs from advicing PA for their patients; (e) the need to establish a PA program in the hospital. The questionnaire was pretested in two stages. First, it was appreciated by two researchers who are experienced with this kind of instrument, then it was administered to twenty participants who were asked to give us their feedback. However, the psychometric properties of the instrument were not measured.

**Statistical analysis:** the results were transcribed into an Excel spreadsheet. The questionnaire was removed from the analysis in case of missing data related to knowledge. All analyses were conducted using SPSS (v.22). Descriptive statistics were used to describe all of data. Inferential statistics were performed to find associations. The associations between knowledge of the PA benefits and different demographic and professional variables were analyzed using Chi-squared test. The differences between the views of physicians and nurses were analyzed using Mann-Withney test. Significant differences were identified using two-sided P-values of<0.05.

**Ethical considerations:** the study was approved by the Joint Ethics Committee of the Faculty of Medicine and Pharmacy at Mohammed V University of Rabat, Morocco (November, 2019; CRTN: 104/19). All participants provided informed consent before participating.

## Results

**Figure 1:**
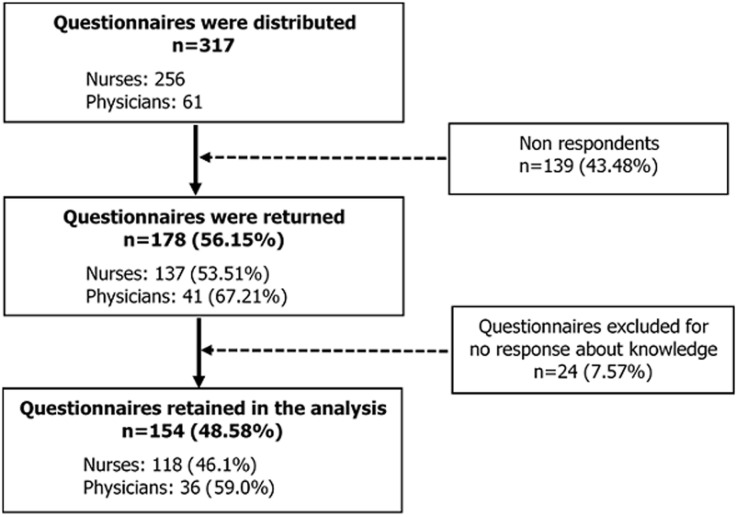
diagram of survey participation

Among 317 questionnaires distributed, a total of 154 questionnaires were retained in the analysis (118 nurses and 36 physicians). The participation rate was 48.58% (59.0% for physicians and 46.1% for nurses). In Figure 1, the information about the survey participation is illustrated.

**Demographic and professional information:** in Table 1, demographic and professional information are presented (gender, department, profession, duration of oncology experience and Knowledge about the PA benefits in oncology). In brief, the majority of participants are female (64.9%) and nurses (76.6%). Also, the majority of participants (80%) declared to be informed about PA benefits in oncology. The knowledge rate seems to be associated with the nature of the profession practiced, so the proportion of physicians informed about PA benefits in oncology seems to be greater than that of nurses (Chi-squared test, p = 0.016) (Table 1).

**Table 1:** description of participants (N=154) and associations between demographic and professional characteristics and their knowledge about the physical activity benefits in oncology

Characteristics	Frequency n (%)	Knowledge about the PA benefits in Oncology		p-value*
		Yes n (%)	No n (%)	
**Gender**				0.825
Male	54 (35.1)	44 (81.5)	10 (18.5)	**-**
Female	100 (64.9)	80 (0.8)	20 (0.2)	**-**
Total	154	124 (80.5)	30 (19.5)	**-**
**Department**				0.283
Visiting	23 (15.1)	19 (82.6)	4 (17.4)	**-**
Hospitalization	70 (46.1)	56 (80.0)	14 (20.0)	**-**
Gynecology	22 (14.5)	21 (95.5)	1 (4.5)	**-**
Other	37 (24.3)	28 (75.7)	9 (24.3)	**-**
Total	152	124 (81.6)	28 (18.4)	**-**
**Profession**				**0.016**
Physician	36 (23.4)	34 (94.4)	2 (5.6)	**-**
Nurse	118 (76.6)	90 (76.3)	28 (23.7)	**-**
Total	154	124 (80.5)	30 (19.5)	**-**
**Duration of experience**				0.723
<5 years	57 (37.0)	47 (82.5)	10 (17.5)	**-**
5-10 years	67 (43.5)	52 (77.6)	15 (22.4)	**-**
>10 years	30 (19.5)	25 (83.3)	5 (16.7)	**-**
Total	154	124 (80.5)	30 (19.5)	**-**

* Chi-squared test

**Figure 2:**
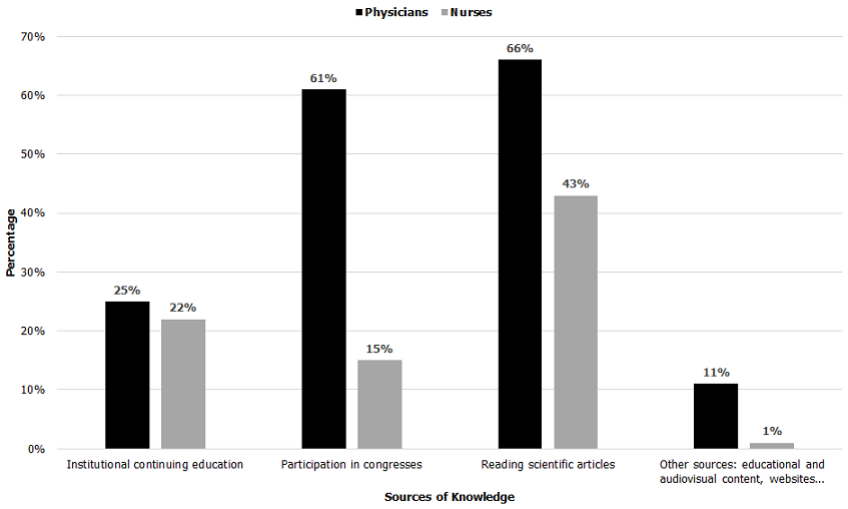
sources of knowledge on physical activity in oncology care

**Sources of knowledge on PA in oncology care:** Figure 2 shows that the source of knowledge on PA in oncology care most mentioned by participants is reading scientific articles, while only a minority of them retained the institutional continuing education and other sources (educational and audiovisual content, websites). Among the majority of physicians, participation in congresses (61.1%) represents a main source of their knowledge, but it seems to be less important for nurses (15%) (Figure 2).

**Figure 3:**
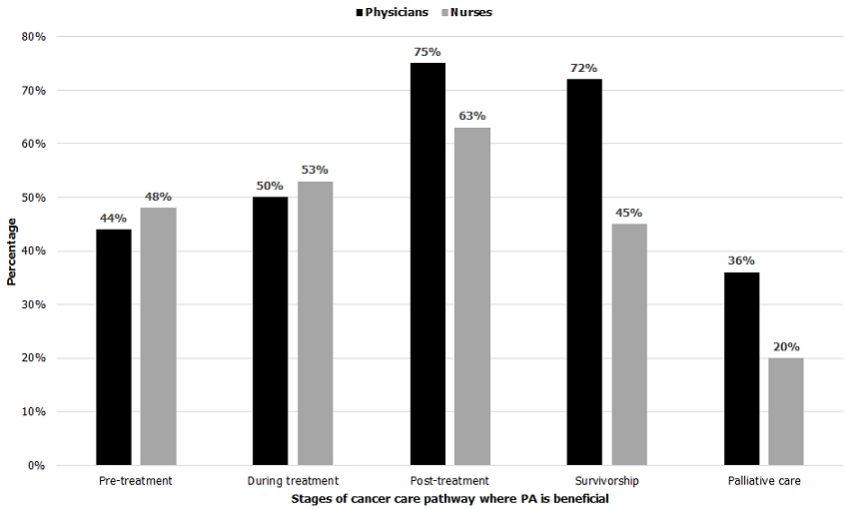
stages of cancer care pathway where physical activity is beneficial

**Stages of cancer care pathway where PA is beneficial:** Figure 3 shows that the majority of participants thought that PA is beneficial in post-treatment and as a measure to be maintained in order to improve survival. The majority did not find it beneficial in the palliative care. About half of the study participants found PA to be beneficial before and during treatment. Physicians would seem to be more favorable (72%) than nurses (45%) with the improvement of survival by increasing PA (Figure 3).

**Views about PA in oncology care:** Table 2 shows that the majority of HCPs views seem to strongly agree or agree with the all statements. The participants seem to be convinced of the positive perception of their collaborators (physicians and nurses) on the improvement of the quality of life and the survival of the CS by PA. Participants thought patients think they should stay physically active, and they affirmed that their patients ask for permission to exercise. Also, participants would appear to be confident that the PA advice will be well received by patients, and therefore it will be carried out by them. According to the participants, family and friends encourage patients to follow the PA advice. Nonetheless, clinicians have recognized that some clinical factors prevent them from recommending PA for their patients. Except one item, no statistically significant difference was shown between the views of physicians and nurses. However, nurses would seem to be more favorable with establishment of PA program at the hospital (Mann-Withney test, p = 0.04) (Table 2).

**Table 2:** views of physicians and nurses about physical activity advice in oncology care

Items	HCPs	1 n(%)	2 n(%)	3 n(%)	4 n(%)	5 n(%)	p-value*
Physicians believe that PA improves the quality of life and survival	Physicians	24 (23.8)	4 (19.0)	5 (26.3)	2 (40.0)	1 (14.3)	0.99
	Nurses	77 (76.2)	17 (81.0)	14 (73.7)	3 (60.0)	6 (85.7)	
	Total	101 (66.0)	21 (13.7)	19 (12.4)	5 (3.3)	7 (4.6)	
Nurses believe that PA improves quality of life and survival	Physicians	31 (22.5)	3 (42.9)	1 (20.0)	0 (0.0)	1 (50.0)	0.45
	Nurses	107 (77.5)	4 (57.1)	4 (80.0)	2 (100.0)	1 (50.0)	
	Total	138 (89.6)	7 (4.5)	5 (3.2)	2 (1.3)	2 (1.3)	
Patients believe they should stay physically active	Physicians	21 (27.3)	5 (20.8)	3 (13.6)	3 (13.6)	4 (50.0)	0.44
	Nurses	56 (72.7)	19 (79.2)	19 (86.4)	19 (86.4)	4 (50.0)	
	Total	77 (50.3)	24 (15.7)	22 (14.4)	22 (14.4)	8 (5.2)	
Patients ask for medical advice in order to exercise	Physicians	18 (20.0)	8 (38.1)	4 (16.0)	2 (66.7)	4 (28.6)	0.30
	Nurses	72 (80.0)	13 (61.9)	21 (84.0)	1 (33.3)	10 (71.4)	
	Total	90 (58.8)	21 (13.7)	25 (16.3)	3 (1.9)	14 (9.1)	
Providing PA advice is generally well received by patients	Physicians	22 (24.4)	7 (23.3)	5 (25.0)	0 (0.0)	2 (40.0)	0.88
	Nurses	68 (75.6)	23 (76.7)	15 (75.0)	5 (100.0)	3 (60.0)	
	Total	90 (60.0)	30 (20.0)	20 (13.3)	5 (3.3)	5 (3.3)	
Patients follow the PA advice given by Physicians and nurses	Physicians	19 (28.4)	12 (26.7)	1 (5.0)	4 (33.3)	0 (0.0)	0.09
	Nurses	48 (71.6)	33 (73.3)	19 (95.0)	8 (66.7)	9 (100.0)	
	Total	67 (43.8)	45 (29.4)	20 (13.1)	12 (7.5)	9 (5.9)	
Families and friends of patients encourage PA	Physicians	20 (29.9)	5 (17.2)	7 (28.0)	3 (18.8)	1 (6.7)	0.09
	Nurses	47(70.1)	24 (82.8)	18 (72.0)	13 (81.3)	14 (93.3)	
	Total	67 (44.1)	29 (19.1)	25 (16.4)	16 (10.5)	15 (9.9)	
I need more information to advice PA for patients	Physicians	27 (23.7)	2 (18.2)	2 (22.2)	2 (28.6)	3 (27.3)	0.93
	Nurses	87 (76.3)	9 (81.8)	7 (77.8)	5 (71.4)	8 (72.7)	
	Total	114 (75.0)	11 (7.2)	9 (5.9)	7 (4.6)	11 (7.2)	
Some patient factors that prevent me from advising PA: fatigue (...)	Physicians	24 (21.8)	7 (26.9)	0 (0.0)	1 (25.0)	4 (66.7)	0.31
	Nurses	86 (78.2)	19 (73.1)	6 (100.0)	3 (75.0)	2 (33.3)	
	Total	110 (72.4)	26 (17.1)	6 (3.9)	4 (2.6)	6 (3.9)	
It is necessary to have PA program at the hospital	Physicians	27 (20.9)	2 (20.0)	1 (14.3)	1 (100.0)	5 (83.3)	**0.04**
	Nurses	102 (79.1)	8 (80.0)	6 (85.7)	0 (0.0)	1 (16.7)	
	**Total**	129 (84.3)	10 (6.5)	7 (4.6)	1 (0.6)	6 (3.9)	

**1)** Strongly agree; **2)** agree; **3):** neither agree or disagree**; 4):** disagree**; 5):** strongly disagree; (*): Mann-Withney test; (...) Other patient factors proposed for participants: advanced cancer, nausea, lymphedema, leukopenia, thrombocytopenia, cachexia

## Discussion

**Healthcare professionals´ knowledge about PA benefits in oncology:** in our study, while the nature of the profession practiced seems to be associated with knowledge about the PA benefits for PC (physicians seem to be more informed than nurses), other socio-professional characteristics does not. In previous studies, some HCPs´ characteristics have been related to PA´ knowledge such as experience [10] and specialty in oncology [11]. In fact, the PA has several benefits for the patient in the all cancer care pathway. The majority of participants recognized these benefits in post-treatment, and about the half recognized them before and during treatment. Several systematic reviews with meta-analysis demonstrated that PA improves the quality of life, when it is programmed after treatment [12,13] and during treatment [14-16]. When introduced before treatment, the PA prevents postoperative complications and improves physical functions [17-19]. However, few participants agreed to the PA benefits during the palliative care. However, the PA appears to improve physical functions and quality of life for PC in this stage [20-22]. The majority of participants recognized the PA benefits on survivorship, especially physicians. A meta-analysis has shown that regular PA can prevent cancer relapse and improve survival [23]. However, according to international guidelines, there is still a lack of scientific evidence that PA improve the survival [2].

**Healthcare professionals´ views about PA safety in oncology:** the safety of the PC was accused as a barrier to the PA advice by the majority of participants. This barrier has also been exposed in several surveys [11,24-26]. Nevertheless, the safety of the PA intervention was supported by sufficient scientific evidence [2]. It remains safe even in advanced cancers [27]. The majority of participants considered certain cancer side effects - wrongly - as a barrier to the PA advice. However, these side effects may be decreased by PA, such as fatigue [28] and nausea [29]; or without increasing like secondary lymphedema [30]. From another perspective, the distrust of participants is not unjustified. Indeed, many RCTs on PA in advanced cancers have shown a poor safety report [27]. However, the risk of PA adverse events remains low [31]. In addition, the HCPs find the lack of empirical data that can serve as a reference in PA advancing [32]. Uncertainty of HCPs about clinical factors may impact their PA promoting behavior, current finding underscore the need for further clinical and empirical research [11].

**Lack of information and other barriers to PA advice:** while the majority of participants said they learned about the PA benefits, they thought they need more information in order to recommend PA for their patients. This statement could be justified by the lack of information on one hand [9,33,34], and on the other hand to the lack of training opportunities [6]. The institutional continuing education and participation in congresses needs to be further developed as training opportunities about PA advice, especially among nurses. This lack of knowledge did not prevent participants from having favorable views about PA in oncology. In the same way, HCPs have shown positive views on the PA benefits in oncology [6,8,35-37]. Several studies have examined the practice of PA advice by HCPs in oncology. A study found that although HCPs recognized the PA benefits, only few of them actually advice it for PC [10]. In another study, the majority of nurses mostly recommended PA during treatment, compared with half of them who recommended it in all cancer care pathway [36]. Consequently, the lack of information related to the PC security cannot alone explain the lack of the practice of PA advice. Actually, there are several barriers to the PA advising: barriers related to HCPs such as lack of information, barriers related to perception of patients and institutional or structural barriers [33]. The lack of PA program at the hospitals of the participants could be considered as a structural barrier, based on the views of the participants, especially oncologist nurses. In the same way, the most of RCTs who have shown the PA benefits in post-treatment have used supervised interventions that were specific in their frequencies, intensities and durations [12].

**Healthcare professionals views about Patient adherence to the PA advice and the role of the family:** the majority of participants thought that patient follow the PA advice given by HCPs. Nonetheless, the opposite has been revealed [38]. Also, while some studies have shown that family and friends advised PC to rest and avoid PA [37,39]; but study participants thought the opposite. Correspondingly, the Moroccan context revealed that the family plays an important role in the psychological support of the PC [40]. In view of the foregoing, standardization with PA advice in oncology in the Moroccan context requires the dismantling of above mentioned barriers. In this way, starting with the continuing education of both physicians and nurses about PA advice would be an appropriate measure to plan. In addition, it is important to adopt a holistic model and family-centered approach to oncology care.

**Limitations:** this study has several limitations that should be acknowledged. First, given the cross-sectional design of the study limits the establishment of causal associations between the participants´ characteristics, and their knowledge/views on PA in oncology. Second, the self-assessment using the survey systematically constitutes a bias related to the recall. Third, the results should be taken with moderation because the psychometric properties of the questionnaire were not measured. Fourth, to provide socially accepted answers would have led the participants to show their agreement with some statements, that is why the results should be interpreted with caution. In addition, the results of this survey carried out at National Institute of Oncology cannot be generalized across or outside the country, because each establishment has its own characteristics in skills and professional practices.

## Conclusion

The present study remains - to the authors' knowledge - the first study which explored the knowledge and views of HCPs on the PA advice in oncology in Morocco. The results showed that the majority of participants recognized the benefits of PA, and they expressed positive views on the PA advice. Physicians seem to be more informed than nurses about the PA benefits in oncology care, while nurses seem to agree more with the establishment of a PA program in hospitals. The majority of participants thought PA is not beneficial to PC in the palliative care. In addition, they believed that some clinical factors related to PC constitute a barrier to the PA advice. However, participants stay confident about patients' adherence to the PA advice and in the encouragement to follow these recommendations by the family and friends. We suggest to scientific societies and decision-makers to strengthen training actions in the PA advice in order to meet the needs of HCPs in oncology.

### What is known about this topic

- *There is strong scientific evidence to support the benefits of physical activity on physical functions and quality of life for patient;*
- *Some socio-professional characteristics have been associated with knowledge of healthcare professionals about benefits of physical activity, such as experience and specialty in oncology;*
- *There are several barriers to the physical activity advising: barriers related to healthcare professionals such as lack of information, barriers related to perception of patients and institutional or structural barriers*.

### What this study adds

- *A difference in the knowledge rate about benefits of physical activity in oncology between oncologist physicians and nurses. Physicians seem to be more informed than nurses;*
- *Nurses seem to agree more than physicians with the establishment of a specific physical activity program in hospitals;*
- *Some clinical factors related to the patient constitute a barrier of physical activity advising for our participants including palliative care*.

## References

[1] Fitzmaurice C, Abate D, Abbasi N, Abbastabar H, Abd-Allah F, Abdel-Rahman O (2019). Global, regional, and national cancer incidence, mortality, years of life lost, years lived with disability, and disability-Adjusted life-years for 29 cancer groups, 1990 to 2017: a systematic analysis for the global burden of disease study. JAMA Oncol.

[2] Segal R, Zwaal C, Green E, Tomasone JR, Loblaw A, Petrella T (2017). Exercise for people with cancer: a clinical practice guideline. curr oncol.

[3] Loprinzi PD, Lee H (2014). Rationale for promoting physical activity among cancer survivors: literature review and epidemiologic examination. Oncol Nurs Forum.

[4] Jones LW, Courneya KS, Fairey AS, Mackey JR (2004). Effects of an oncologist's recommendation to exercise on self-reported exercise behavior in newly diagnosed breast cancer survivors: a single-blind, randomized controlled trial. Ann Behav Med.

[5] Fisher A, Williams K, Beeken R, Wardle J (2015). Recall of physical activity advice was associated with higher levels of physical activity in colorectal cancer patients. BMJ.

[6] Douglas F, Torrance N, Van Teijlingen E, Meloni S, Kerr A (2006). Primary care staff's views and experiences related to routinely advising patients about physical activity; a questionnaire survey. BMC Public Health.

[7] Sheill G, Guinan E, O Neill L, Hevey D, Hussey J (2018). Physical activity and advanced cancer: the views of chartered physiotherapists in Ireland. Physiother Theory Pract.

[8] Hardcastle SJ, Kane R, Chivers P, Hince D, Dean A, Higgs D (2018). Knowledge, attitudes, and practice of oncologists and oncology health care providers in promoting physical activity to cancer survivors: an international survey. Support Care Cancer.

[9] Haussmann A, Ungar N, Gabrian M, Tsiouris A, Sieverding M, Wiskemann J (2018). Are healthcare professionals being left in the lurch? The role of structural barriers and information resources to promote physical activity to cancer patients. Support Care Cancer.

[10] Spellman C, Craike M, Livingston P (2014). Knowledge, attitudes and practices of clinicians in promoting physical activity to prostate cancer survivors. Health Educ J.

[11] Tsiouris A, Ungar N, Haussmann A, Sieverding M, Steindorf K, Wiskemann J (2018). Health care professionals' perception of contraindications for physical activity during cancer treatment. Front Oncol.

[12] Fong DYT, Ho JWC, Hui BPH, Lee AM, Macfarlane DJ, Leung SSK (2012). Physical activity for cancer survivors: meta-analysis of randomised controlled trials. BMJ.

[13] Buffart LM, Kalter J, Sweegers MG, Courneya KS, Newton U, Aaronson NK (2017). Effects and moderators of exercise on quality of life and physical function in patients with cancer: an individual patient data meta-analysis of 34 RCTs. Cancer Treat Rev.

[14] Furmaniak AC, Menig M, Markes MH (2016). Exercise for women receiving adjuvant therapy for breast cancer. Cochrane Database Syst Rev.

[15] Baumann FT, Zopf EM, Bloch W (2012). Clinical exercise interventions in prostate cancer patients-a systematic review of randomized controlled trials. Support Care Cancer.

[16] Gardner JR, Livingston PM, Fraser SF (2014). Effects of exercise on treatment-related adverse effects for patients with prostate cancer receiving androgen-deprivation therapy: a systematic review. J Clin Oncol.

[17] Cavalheri V, Granger C (2017). Preoperative exercise training for patients with non-small cell lung cancer. Cochrane Database Syst Rev Rev.

[18] Chang JI, Lam V, Patel MI (2016). Preoperative pelvic floor muscle exercise and postprostatectomy incontinence: a systematic review and meta-analysis. Eur Urol.

[19] Hijazi Y, Gondal U, Aziz O (2017). A systematic review of prehabilitation programs in abdominal cancer surgery. Int J Surg.

[20] Dittus KL, Gramling RE, Ades PA (2017). Exercise interventions for individuals with advanced cancer: a systematic review. Prev Med (Baltim).

[21] Jensen W, Baumann FT, Stein A, Bloch W, Bokemeyer C, De Wit M (2014). Exercise training in patients with advanced gastrointestinal cancer undergoing palliative chemotherapy: a pilot study. Support Care Cancer.

[22] Ligibel JA, Giobbie-Hurder A, Shockro L, Campbell N, Partridge AH, Tolaney SM (2016). Randomized trial of a physical activity intervention in women with metastatic breast cancer. Cancer.

[23] Cormie P, Zopf EM, Zhang X, Schmitz KH (2017). The impact of exercise on cancer mortality, recurrence, and treatment-related adverse effects. Epidemiol Rev.

[24] Park JH, Oh M, Yoon YJ, Lee CW, Jones LW, Kim S Il (2015). Characteristics of attitude and recommendation of oncologists toward exercise in South Korea: a cross sectional survey study. BMC Cancer.

[25] Karvinen KH, Mcgourty S, Parent T, Walker PR (2012). Physical activity promotion among oncology nurses. Cancer Nurs.

[26] Haussmann A, Ungar N, Tsiouris A, Sieverding M, Wiskemann J, Steindorf K (2020). The influence of cancer patient characteristics on the recommendation of physical activity by healthcare professionals. Int J Behav Med.

[27] Nadler MB, Desnoyers A, Langelier DM, Amir E (2019). The effect of exercise on quality of life, fatigue, physical function and safety in advanced solid tumor cancers: a meta-analysis of randomized control trials. J Pain Symptom Manage.

[28] Kessels E, Husson O, Der CM Van (2018). The effect of exercise on cancer-related fatigue in cancer survivors: a systematic review and. Neuropsychiatr Dis Treat.

[29] Uster A, Ruehlin M, Mey S, Gisi D, Knols R, Imoberdorf R (2018). Effects of nutrition and physical exercise intervention in palliative cancer patients: a randomized controlled trial. Clin Nutr.

[30] Singh B, Disipio T, Peake J, Hayes SC (2016). Systematic review and meta-analysis of the effects of exercise for those with cancer-related lymphedema. Arch Phys Med Rehabil.

[31] Schmitz KH, Courneya KS, Matthews C, Galva DA, Pinto BM, Irwin ML (2010). American college of sports medicine roundtable on exercise guidelines for cancer survivors. Med Sci Sport Exerc.

[32] Wolin KY, Schwartz AL, Matthews CE, Courneya KS, Schmitz KH (2012). Implementing the Exercise Guidelines for Cancer Survivors. J Support Oncol.

[33] Smith-Turchyn J, Richardson J, Tozer R, McNeely M, Thabane L (2016). Physical activity and breast cancer: a qualitative study on the barriers to and facilitators of exercise promotion from the perspective of health care professionals. Physiother Canada.

[34] Nadler M, Bainbridge D, Tomasone J, Cheifetz O, Juergens RA, Sussman J (2017). Oncology care provider perspectives on exercise promotion in people with cancer: an examination of knowledge, practices, barriers, and facilitators. Support Care Cancer.

[35] Sheill G, Guinan E, Neill LO, Hevey D, Hussey J (2018). Physical activity and advanced cancer: the views of oncology and palliative care physicians in Ireland. Ir J Med Sci.

[36] Van Veen MR, Hoedjes M, Versteegen JJ, Van De Meulengraaf-Wilhelm N, Kampman E, Beijer S (2017). Improving oncology nurses' knowledge about nutrition and physical activity for cancer survivors. Oncol Nurs Forum.

[37] O´Hanlon E, Kennedy N (2014). Exercise in cancer care in Ireland: a survey of oncology nurses and physiotherapists. Eur J Cancer Care (Engl).

[38] Ottenbacher A, Yu M, Moser RP, Phillips SM, Alfano C, Perna FM (2015). Population estimates of meeting strength training and aerobic guidelines, by gender and cancer survivorship status: Findings from the health information national trends survey (HINTS). J Phys Act Heal.

[39] Donnelly CM, Lowe-strong A, Rankin JP, Campbell A, Allen JM, Gracey JH (2010). Physiotherapy management of cancer-related fatigue: a survey of UK current practice.

[40] Errihani H, Mrabti H, Sbitti Y, Kaikani W, El Ghissassi I, Afqir S (2010). Psycho-social and religious impact of cancer diagnosis on Maroccans patients: Experience from the national oncology center of Rabat. Bull Cancer.

